# Delay discounting in children exposed to disaster

**DOI:** 10.1371/journal.pone.0243994

**Published:** 2020-12-30

**Authors:** Yusuke Matsuyama, Takeo Fujiwara, Yasuyuki Sawada, Junko Yagi, Hirobumi Mashiko, Ichiro Kawachi, for the Great East Japan Earthquake Follow-up for Children Study Team

**Affiliations:** 1 Department of Global Health Promotion, Tokyo Medical and Dental University, Tokyo, Japan; 2 The University of Tokyo, Tokyo, Japan; 3 Asian Development Bank, Mandaluyong, Philippines; 4 Department of Psychiatry, Iwate Medical University, Iwate, Japan; 5 Fukushima Rehabilitation Center for Children, Fukushima, Japan; 6 Department of Social and Behaviroral Science, Harvard School of Public Health, Boston, Massachusetts; University of the West Indies at Saint Augustine, TRINIDAD AND TOBAGO

## Abstract

Delay discounting is an important predictor of future health and academic success in children but can change in environmental uncertainty situations. Here we show that the experience of loss of housing in the Great East Japan Earthquake 2011—but not other psychological trauma such as loss of loved ones—was correlated delay discounting of children. In 2014, we assessed delay discounting in children (N = 167; mean age = 8.3 years-old), who were preschool age at the time of the earthquake (mean age at the time of disaster = 4.8 years-old) in a time-investment exercise where children allocated five tokens between rewards "now" (one candy per token on the same day) versus "one month later" (two candies per token one month later). The number of tokens allocated for "now" was higher by 0.535 (95% confidence interval: −0.012, 1.081) in children who had their housing destroyed or flooded than those with no housing damage. Other types of traumatic experiences were not associated with delay discounting.

## Introduction

Delay discounting–i.e., the degree to which individuals choose between smaller, immediate rewards versus larger, later rewards—is related to goal-oriented behavior, predicting outcomes in life, such as academic performance [1], and health maintenance behaviors [1,2]. In Mischel’s marshmallow study, delayed gratification—i.e., putting off the consumption of a smaller, immediate reward in favor of a larger, delayed reward—was measured by the duration of time that preschool children could resist an immediate, smaller reward (one marshmallow) versus a delayed large reward (two marshmallows) [3,4]. Over long-term follow-up, children with longer wait times were shown to achieve higher SAT scores [5], higher social competences in adolescence [6], and even lower body-mass index (BMI) in adulthood [7]. The ability to delay gratification develops during childhood, which can be negatively influenced by childhood adversity, such as poverty and maltreatment [8–10]. While delayed gratification represents behavior inhibition, delay discounting measures choice preference and is suggested to reflect a higher-order cognitive process of subjective evaluation of rewards under uncertainty [11].

Environmental uncertainty at the time of decision-making may influence delay discounting. For example, a replication of Mischel’s experiment found that children’s delay times were much shorter when conducted with an unreliable experimenter—i.e., someone who failed to keep a promise to the children prior to the experiment—compared to a reliable experimenter [12]; suggesting that delay discounting of children increased under conditions of environmental uncertainty. This is in line with Mullanaithan & Shafir’s scarcity hypothesis, which posits that poverty (scarcity) is a condition of environmental uncertainty; that when individuals are placed in situations of scarcity, they develop present bias [13].

In this paper, we examined the impact of disasters on delay discounting in a field experiment among children who were exposed to the Great East Japan Earthquake in 2011. Disaster damage causes scarcity (loss of property) and the extreme shock can disturb people’s perceptions about the reliability of the world. Previous studies reported that disasters could influence the behavioral preferences of grown-ups [14–16]. In the case of the Great East Japan Earthquake, Akesaka employed nationwide panel data before and after the disaster and reported that present bias or hyperbolic discounting—the extent that discounting rate becomes steeper when it comes to choices in the near future (so-called β discounting)–increased for adults living in tsunami-damaged municipalities while discount factor consistent over time or exponential discounting (δ discounting) did not significantly change [15]. Sawada et al. also reported that the severity of housing damage in the disaster was associated with stronger present bias among older people [14,16]. A similar association was observed among farmers whose house was damaged by the Philippines’ serious floods in 2012 [17]. Yet, to the best of our knowledge, no study has investigated the impact of disasters on delay discounting in children who experience the trauma in preschool age, which is a sensitive period to develop the ability.

## Methods

### Study participants

We used the data from the Great East Japan Earthquake Follow-up for Children (GEJE-FC) study. The sampling design and the detail of the GEJE-FC study are reported elsewhere [18]. In brief, children affected by the Great East Japan Earthquake and tsunami at the age of 3–5 years old and their caregivers were recruited from three affected areas (Iwate, Miyagi, and Fukushima prefectures). The baseline survey was conducted from September 2012 to June 2013 (around one and a half to two years after the earthquake), and the participants were followed annually. Written informed consent was obtained from all participants; parents or legal guardians provided consent for children. The consent procedures and instruments of this study were approved by the Research Ethics Committee at the National Center for Child Health and Development in Tokyo, Japan.

Information on disaster-related traumatic experiences of children was collected in the baseline survey. Delay discounting was assessed in the third follow up survey in 2014. Of the 179 children who participated in the follow-up survey, 12 were excluded because of a lack of data on the delay discounting. Accordingly, the data on 167 children (boy = 50.9%) was analyzed (S1 Fig). The baseline characteristics were not significantly different between children providing and not proving the follow-up data (S1 Table). The mean ages of children were 4.8 years old at the time of the disaster and 8.3 years-old when their delay discounting was measured.

### Measurements

Delay discounting was elicited in 2014 by an incentivized experiment using tokens developed by Angerer et al. [19]. The child was presented with options of "get the *same* number of candies *now* as placed tokens" and "get *twice* a number of candies *one month later* as placed tokens;" and asked to allocate five tokens between the two options. For example, if a child allocates two tokens for *“now”* and three tokens for *“one month later,”* the child gets two candies now and six candies a month later; thus, in total, she/he gets eight candies. On the other hand, if a child allocates four tokens for *“now”* and one token for *“one month later,”* the child gets four candies now and two candies one month later; thus, in total, she/he gets six candies (S2 Fig). Therefore, the larger number of tokens allocated for *“now”* represents the child more discount future reward because she/he preferred a smaller-sooner reward than a larger-later one. We used the number of tokens allocated for *“now”* as the dependent variable in the analysis.

Caregivers were asked to report the extent of housing damage, with response options of *“no damage*,*” “partial*,*” “minor*,*” “major*,*” “destroyed*,*” and “flooded”*; we categorized this variable into "no damage," "partly damaged (including *partial*, *minor* and *major*), " and "destroyed or flooded" in the analysis. The variable was used with a categorical scale. Further, severe traumatic experiences of children were assessed through semi-structured interviews by child psychiatrists or clinical psychologists. We collected information on 1) separation from the caregiver, 2) loss of close family members or relatives, 3) loss of distant relative or friend, 4) witnessing tsunami waves, 5) witnessing a fire, 6) witnessing someone being swept away by the tsunami, and 7) seeing a dead body, based on a previous study assessing children’s mental health after a tsunami [20]. We have collected the information on experiences related to the nuclear power plant failure; however, it was not used in this study as the experience was concentrated in participants from Fukushima prefecture. The interviewers did not probe further when the child did not remember their experiences during the disaster, as doing so might have triggered painful, traumatic memories. Each traumatic experience and its total numbers were used as the independent variable.

The characteristics of children, age, sex, socioeconomic status of the family, and traumatic experiences before the disaster were assessed by questionnaires to caregivers in the baseline survey. The socioeconomic status of the family before the disaster was assessed by questionnaire. The caregivers answered about maternal educational attainment (*“high school or less*,*” “some college*,*”* and *“college or more”*), parental occupation and employment status (*“non-manual worker*,*” “manual worker*,*”* and *“unemployed”*), household subjective economic status before the disaster (*“not stable*,*” “fairly stable*,*”* and *“stable”*), and household income at the follow-up (<3.0, 3–5.9, ≥6.0 million JPY; 1 USD ≈ 110 JPY). Exposure to traumatic experiences before the disaster was also assessed by the questionnaire. The caregivers answered whether the child had the following experiences: 1) involved in a serious accident, 2) witnessed a serious accident, 3) attacked by a dog or other animals, 4) had a close friend or family member who had a serious illness, 5) death of a close friend or family member, 6) visited the hospital due to serious disease or injury, or underwent a serious medical procedure, or admitted to hospital, 7) separated from a caregiver; experienced sexual assault, 8) experienced other criminal assault 9) bullied by peers at preschool or in the neighborhood 10) experienced violence from a close friend or family member, 11) witnessed a violent incident involving a close friend or family member, 12) had a close friend or family member who attempted suicide, 13) experienced a previous natural disaster, and 14) other stressful events.

### Analysis

A baseline balancing test to check the randomness of housing damage was performed by regressing housing damage on a set of children’s background before the disaster, that is, the subjective economic status of the family and the child’s traumatic experiences before the disaster.

In the main analysis, multiple linear regression models were fitted to investigate the association between the number of tokens allocated for *“now”* (i.e., greater delay discounting) and housing damage, traumatic experiences, or its total numbers, adjusted for children’s age and sex. To consider the socioeconomic status of families, household subjective economic status before the disaster and maternal education were adjusted for. Finally, household income at the follow-up survey was added to the model to examine whether the current economic situation explains the association between disaster damage and child’s delay discounting. Of the 167 children, 50 children were multiply involved from 25 families (i.e., 2 children from a family); thus, standard error clustering within the household was estimated to consider within-family correlation. Missing information on categorical independent variables was included as dummy variables.

## Results

**Fig 1:**
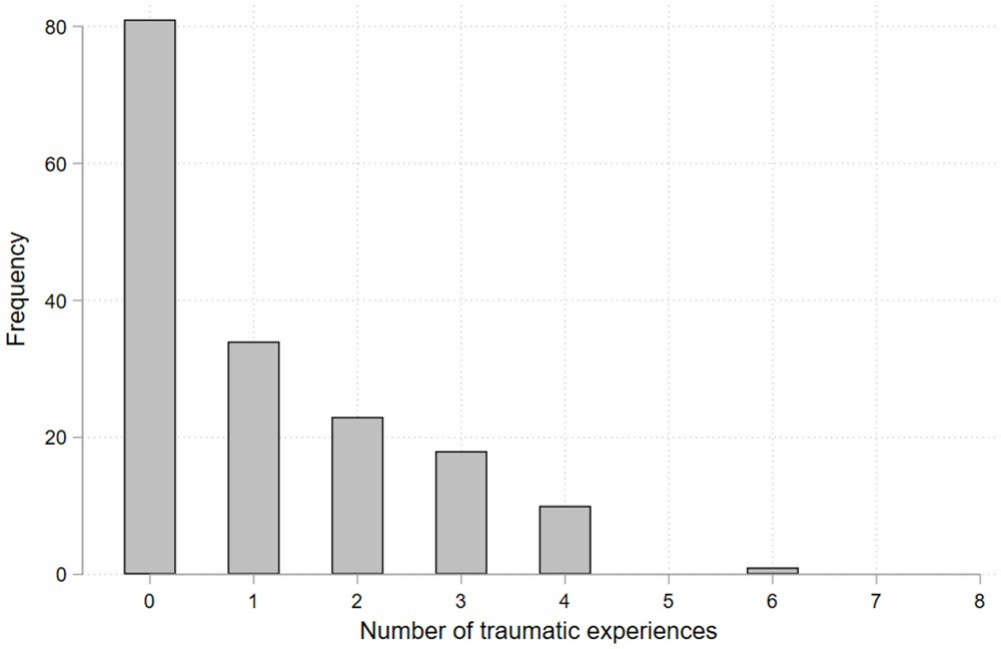
Distribution of the number of traumatic experiences (N = 167).

**Fig 2:**
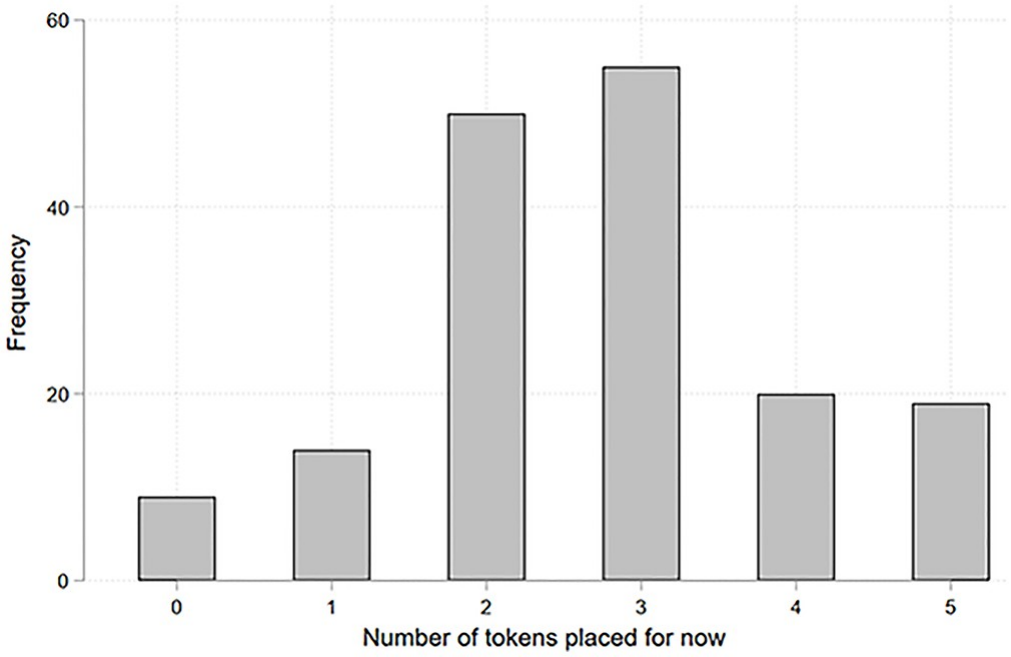
Distribution of the number of tokens placed for “now” (N = 167).

Table 1 shows the demographic characteristics of children. Housing damage was experienced by 43%. Apart from housing damage, the most frequent traumatic experience was witnessing tsunami waves (28%), followed by separation from the caregiver (23%) and witnessing a fire (13%). More than half of the children had experienced one or more traumatic events at the disaster (Fig 1). As Fig 2 shows, 62.8% of children allocated two or three tokens for "*now*," with an average of 2.7 tokens (SD = 1.3). The baseline balancing test showed that housing damage was not associated with children’s pre-disaster backgrounds (S2 Table), indicating that housing damage was exogenously and randomly assigned.

**Table 1:** Demographic characteristics of children (N = 167).

	n/mean	%/SD
Number of tokens placed for "now"	2.7	1.3
Child’s age	8.3	1.4
Child’s sex		
Boy	85	50.9%
Girl	82	49.1%
Traumatic experience at the Great East Japan Earthquake		
Loss of housing property		
No damage	90	53.9%
Partly damaged	32	19.2%
Destroyed/flooded	35	21.0%
Missing	10	6.0%
Separation from caregiver		
No	88	52.7%
Yes	39	23.4%
Missing	40	24.0%
Lost close family member or relative		
No	99	59.3%
Yes	12	7.2%
Missing	56	33.5%
Lost distant relative or friend		
No	90	53.9%
Yes	14	8.4%
Missing	63	37.7%
Witnessed tsunami waves		
No	85	50.9%
Yes	46	27.5%
Missing	36	21.6%
Witnessed a fire		
No	108	64.7%
Yes	21	12.6%
Missing	38	22.8%
Witnessed someone being swept away by the tsunami		
No	123	73.7%
Yes	8	4.8%
Missing	36	21.6%
Saw a dead body		
No	123	73.7%
Yes	5	3.0%
Missing	39	23.4%

Abbreviation: Standard deviation, SD.

**Fig 3:**
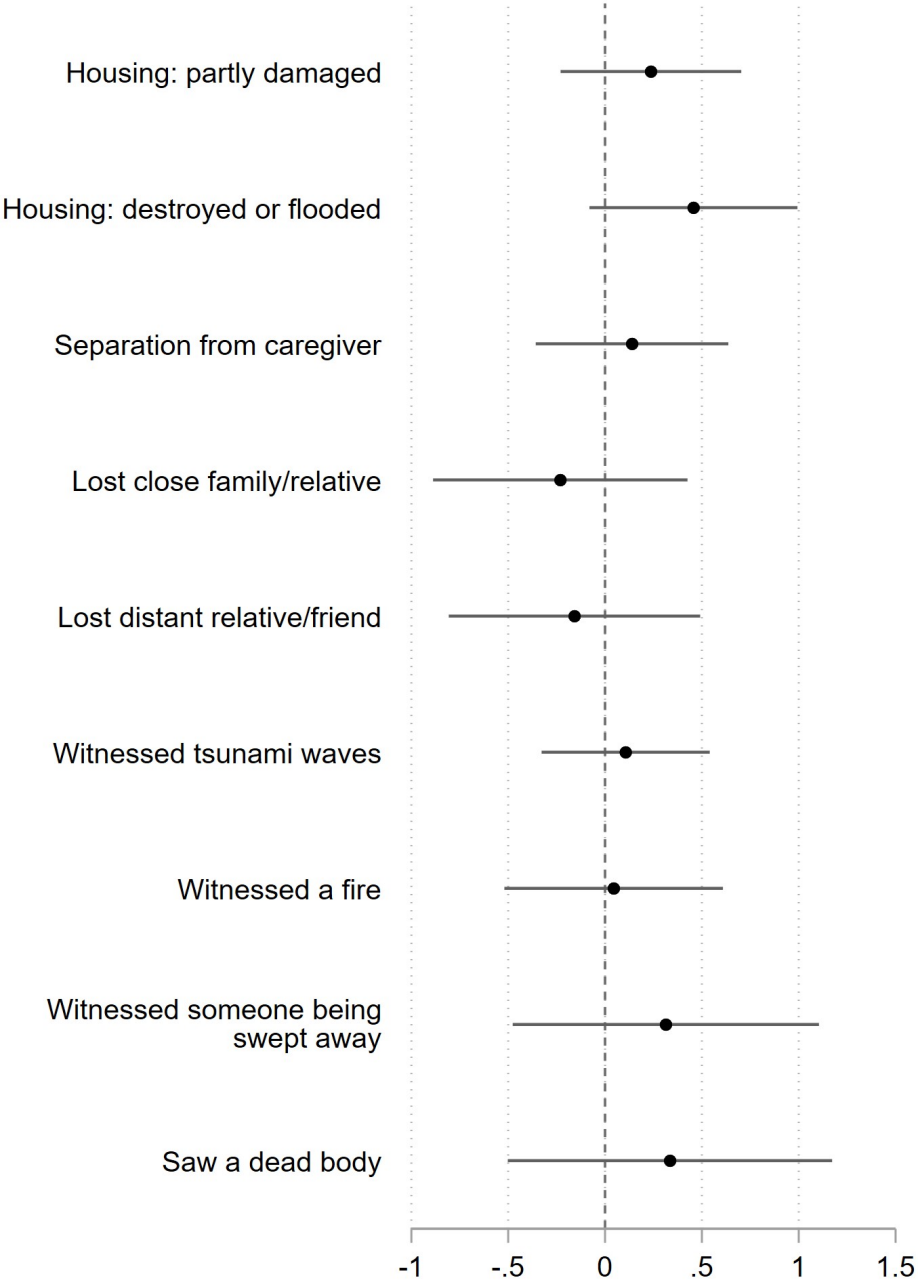
Association between traumatic experiences and delay discounting adjusted for child’s age, sex, maternal education, household subjective economic status before the disaster, and household income at the follow−up survey. Missing values were included as dummy variables, but estimates were not reported; every traumatic experience was separately included (i.e., not mutually adjusted for).

Table 2 shows the association between traumatic experiences at the disaster and delay discounting of children. Children whose house was destroyed or flooded by tsunami showed a higher delay discounting than those without housing property loss after adjusting for child’s age and sex (coefficient = 0.613, 95% confidence interval, CI: 0.045, 1.181). When we also adjust for maternal education and household subjective economic status before the disaster, the association became non-significant, but the point and interval estimates suggested a similar association (coefficient = 0.535, 95% CI: −0.012, 1.081). Household income at the follow-up survey explained 14.8% of the association (Model 3). Other psychological traumas were not associated with delay discounting (Table 2 and Fig 3). Moreover, the total number of traumatic experiences, as shown in Table 3, was not associated with the delay discounting of children.

**Table 2:** Association between traumatic experiences and delay discounting (N = 167).

	Model 1	Model 2	Model 3
	Coef. (95% CI)	Coef. (95% CI)	95% CI (P-value)
Housing damage			
No damage	ref	ref	ref
Partly damaged	0.135 (−0.319, 0.589)	0.218 (−0.252, 0.687)	0.237 (−0.230, 0.703)
Destroyed or flooded	0.613 (0.045, 1.181)	0.535 (−0.012, 1.081)	0.456 (−0.081, 0.993)
Other traumatic experiences (ref. no)			
Separation from caregiver	0.208 (−0.278, 0.695)	0.141 (−0.341, 0.623)	0.139 (−0.358, 0.637)
Lost close family member or relative	−0.137 (−0.866, 0.591)	−0.053 (−0.790, 0.684)	−0.231 (−0.888, 0.426)
Lost distant relative or friend	−0.184 (−0.866, 0.498)	−0.166 (−0.816, 0.484)	−0.158 (−0.807, 0.491)
Witnessed tsunami waves	0.069 (−0.400, 0.538)	0.060 (−0.369, 0.489)	0.106 (−0.328, 0.540)
Witnessed a fire	0.218 (−0.379, 0.814)	0.126 (−0.449, 0.701)	0.044 (−0.520, 0.609)
Witnessed someone being swept away by tsunami	0.412 (−0.522, 1.346)	0.279 (−0.520, 1.077)	0.314 (−0.477, 1.104)
Saw a dead body	0.207 (−0.642, 1.055)	0.124 (−0.767, 1.014)	0.335 (−0.502, 1.173)

The dependent variable is delay discounting of children captured by the number of tokens allocated for “now.” Missing values were included as dummy variables, but estimates were not reported; every traumatic experience variable was separately included (i.e., not mutually adjusted for).

Model 1: Adjusted for child’s age and sex.

Model 2: Model 1 + maternal education and household subjective economic status before the disaster.

Model 3: Model 2 + household income at the follow−up survey.

**Table 3:** Association between the number of traumatic experiences and delay discounting (N = 167).

	Model 1	Model 2	Model 3
	Coef. (95% CI)	Coef. (95% CI)	Coef. (95% CI)
Number of traumatic experiences			
1	0.481 (−0.125, 1.087)	0.401 (−0.229, 1.031)	0.337 (−0.280, 0.954)
2	0.023 (−0.477, 0.524)	0.039 (−0.483, 0.561)	0.025 (−0.471, 0.520)
3	0.229 (−0.460, 0.918)	0.137 (−0.587, 0.860)	0.077 (−0.627, 0.780)
≥4	0.351 (−0.458, 1.160)	0.335 (−0.431, 1.101)	0.268 (−0.460, 0.995)

The dependent variable is delay discounting of children captured by the number of tokens allocated for “now”.

Model 1: Adjusted for child’s age and sex.

Model 2: Model 1 + maternal education and household subjective economic status before the disaster.

Model 3: Model 2 + household income at the follow−up survey.

## Discussion

To the best of our knowledge, this is the first study to find an association between the loss of housing property in a disaster and the impatience of children measured by an incentivized experiment. Loss of housing property increased impatience although not statistically significant, while other psychological traumatic experiences, such as loss of a close family member or relative, did not.

Time preference is not constant but changes over time [21]. Exogenous shocks such as disasters have been previously reported to affect time preference. For example, the 2004 Indian Ocean tsunami increased impatience among survivors [22]. Some possible explanations for this pattern include: first, severe disasters, such as the Great East Japan Earthquake in 2011, cause substantial property damage, thereby heightening the sense of uncertainty about the future (and in turn resulting in discounting of future rewards) [23,24]. Greater uncertainty and resource constraints in the postdisaster context might have partially mediated the association between property damage and delay discounting, given that the association was attenuated by including postdisaster household income in the regression model. Second, stress or negative emotion caused by housing property loss could be another channel because people tend to become more impatient when they feel stress [25] or sadness [26].

In the case of the present study, it should be noted that witnessing life-threatening events of others (e.g., witnessed someone being swept away by the tsunami and saw a dead body) was not associated with delay discounting. The reasons remain unknown; however, it is possible that the impact of the loss of housing continues to affect survivors’ lives for many years after the disaster persistently (e.g., because people are still living in temporary trailer homes for up to 6 years after losing their homes), whereas children recover from the loss of life after a period of grief.

According to the so-called β-δ model, an individual’s time preference consists of two parameters, the extent to which they discount delays more steeply when it comes to choices in the near future (quasi-hyperbolic discounting model or β discounting) or consistently over time (exponential discounting or, equivalently, δ discounting) [27]. The dynamically inconsistent preference, or present bias, has been previously reported to be associated with behaviors such as smoking and unhealthy eating [28,29]. Our single-choice experiment in the present study did not allow us to differentiate between these two discount rates, although previous studies have reported that present bias (β) in adults, but not δ discount rates were affected by the Great East Japan Earthquake [14,15]. The finding of the present study suggests that children who suffered housing loss were more impatient which can be attributed to either or both of these two discount rates.

This study has several limitations. First, our experiment provided only one choice without varying the reward size or delay time between initial vs. later reward in order to reduce the cognitive burden on the participant children. As a result, we were not able to quantify the child’s discount rate or separate the two types of discounting factors. However, the single-choice experiment has previously been reported to show similar results to multiple-choice experiments when repeated among the same children [19]. Some studies provided several options of rewards (e.g., candies, stickers, pencils) and allowed children to chose their favorite one [19]), while candies were the only reward in the present study. Thus, the extent of reinforcement might be small for some children. Second, since we do not have information on children’s discount rates before the disaster, our findings rely on cross-sectional between-person comparisons. Lack of information about the child’s time preferences pre-dating the disaster could induce potential confounding. For example, children with higher impatience might have come from households that were already experiencing conditions of scarcity (e.g., parental unemployment or other types of adversity) prior to the disaster. These households may have also been at higher risk of housing damage. However, the severity of housing damage was not associated with households’ socioeconomic status prior to the disaster (S1 Table). Further, the results did not change after adjusting for household socioeconomic status. Third, the municipalities were not randomly selected from the damaged area. We invited the preschools via convenience sampling [18], which helped to conduct the survey after the disaster. Children who experienced more severe trauma might not have participated in the study, which in turn might result in an underestimation of traumatic experiences. Some children might not have accurately reported their traumatic experiences during the interviews or erased their memories due to psychological adaptation. We checked the agreement of the child’s answers with their caregivers and preschool teachers. In the end, we chose to use the child’s answer because it best represents the child’s experience of trauma. We might have failed to identify the association between traumatic experiences and delay discounting because of the small sample size.

## Conclusions

Greater delay discounting was observed among children whose house was damaged by the Great East Japan Earthquake. This finding suggests that children who were affected by a disaster might have short-sighted behavior.

## Supporting information

S1 Fig Flowchart of the participants. (DOCX)

S2 Fig Instruction and examples of the experiment. (DOCX)

S1 Table Baseline characteristics between children provided and not provided follow−up data. (DOCX)

S2 Table Balancing test for characteristics before the disaster; results from ordered logistic regression analysis. (DOCX)
