# Supplementary figures and images for: Delay discounting in children exposed to disaster
Source: PLoS One. 2020 Dec 30;15(12):e0243994. doi: 10.1371/journal.pone.0243994 (PMC7773199; doi:10.1371/journal.pone.0243994)

S1 Fig. Flowchart of the participants


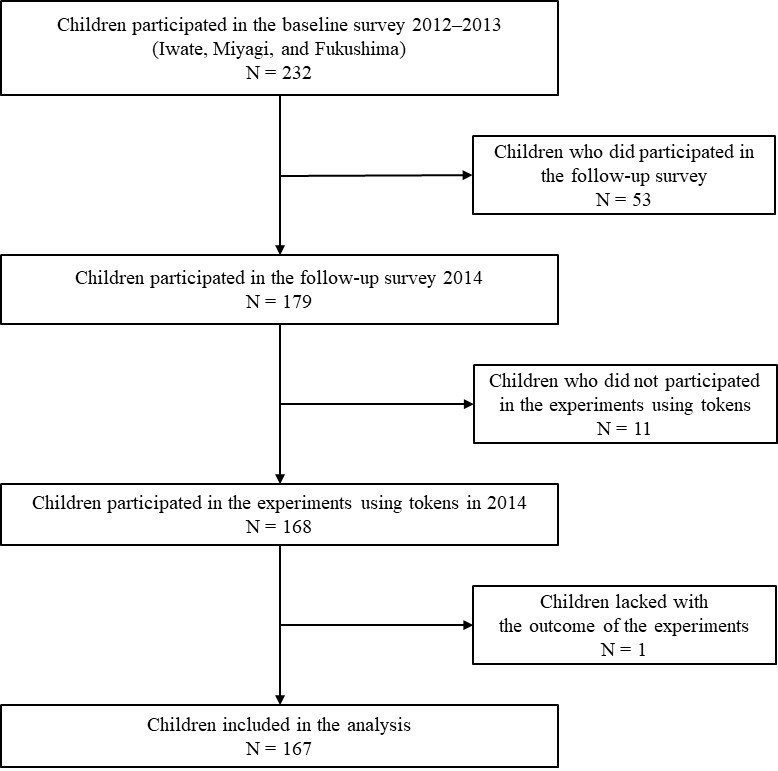

Supplement: S1 Fig — (DOCX) [file pone.0243994.s001.docx]

S2 Fig. Instruction and examples of the experiment


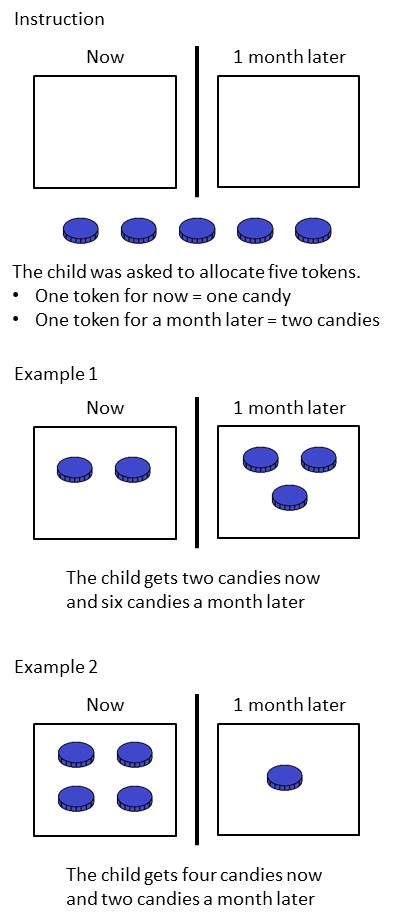

Supplement: S2 Fig — (DOCX) [file pone.0243994.s002.docx]
