# Supplementary material for: Delay discounting in children exposed to disaster
Source: PLoS One. 2020 Dec 30;15(12):e0243994. doi: 10.1371/journal.pone.0243994 (PMC7773199; doi:10.1371/journal.pone.0243994)
Supplement: S1 Table — (DOCX) [file pone.0243994.s003.docx]

**Supporting information**

S1 Table. Baseline characteristics between children provided and not provided follow−up data

|  | Children provided follow−up data (N = 167) | Children not provided follow−up data (N = 12) | P−value |
| --- | --- | --- | --- |
| Child's age at the disaster | 4.8 (1.4) | 5.4 (1.7) | 0.130 ^a^ |
| Child's sex |  |  | 0.620 ^b^ |
| Boy | 85 (50.9%) | 7 (58.3%) |  |
| Girl | 82 (49.1%) | 5 (41.7%) |  |
| Household subjective economic status before the disaster |  |  | 0.230 ^b^ |
| Not stable | 16 ( 9.6%) | 1 ( 8.3%) |  |
| Fairly stable | 62 (37.1%) | 2 (16.7%) |  |
| Stable | 76 (45.5%) | 9 (75.0%) |  |
| Missing | 13 ( 7.8%) | 0 ( 0.0%) |  |
| Mother's education |  |  | 0.630 ^b^ |
| High school or less | 80 (47.9%) | 5 (41.7%) |  |
| Some college | 58 (34.7%) | 5 (41.7%) |  |
| College or more | 16 ( 9.6%) | 2 (16.7%) |  |
| Missing | 13 ( 7.8%) | 0 ( 0.0%) |  |
| Housing damage |  |  | 0.081 ^b^ |
| No damage | 90 (53.9%) | 4 (33.3%) |  |
| Partly damaged | 32 (19.2%) | 6 (50.0%) |  |
| Destroyed or flooded | 35 (21.0%) | 2 (16.7%) |  |
| Missing | 10 ( 6.0%) | 0 ( 0.0%) |  |
| Separation from caregiver |  |  | 0.710 ^b^ |
| No | 88 (52.7%) | 5 (41.7%) |  |
| Yes | 39 (23.4%) | 3 (25.0%) |  |
| Missing | 40 (24.0%) | 4 (33.3%) |  |
| Lost close family member or relative |  |  | 0.480 ^b^ |
| No | 99 (59.3%) | 6 (50.0%) |  |
| Yes | 12 ( 7.2%) | 2 (16.7%) |  |
| Missing | 56 (33.5%) | 4 (33.3%) |  |
| Lost distant relative or friend |  |  | 0.620 ^b^ |
| No | 90 (53.9%) | 6 (50.0%) |  |
| Yes | 14 ( 8.4%) | 2 (16.7%) |  |
| Missing | 63 (37.7%) | 4 (33.3%) |  |
| Witnessed tsunami waves |  |  | 0.820 ^b^ |
| No | 85 (50.9%) | 5 (41.7%) |  |
| Yes | 46 (27.5%) | 4 (33.3%) |  |
| Missing | 36 (21.6%) | 3 (25.0%) |  |
| Witnessed a fire |  |  | 0.110 ^b^ |
| No | 108 (64.7%) | 5 (41.7%) |  |
| Yes | 21 (12.6%) | 4 (33.3%) |  |
| Missing | 38 (22.8%) | 3 (25.0%) |  |
| Witnessed someone being swept away by tsunami |  |  | 0.730 ^b^ |
| No | 123 (73.7%) | 9 (75.0%) |  |
| Yes | 8 ( 4.8%) | 0 ( 0.0%) |  |
| Missing | 36 (21.6%) | 3 (25.0%) |  |
| Saw a dead body |  |  | 0.830 ^b^ |
| No | 123 (73.7%) | 9 (75.0%) |  |
| Yes | 5 ( 3.0%) | 0 ( 0.0%) |  |
| Missing | 39 (23.4%) | 3 (25.0%) |  |

a P−value from t−test

b P−value from chi−square test
