# Supplementary material for: Delay discounting in children exposed to disaster
Source: PLoS One. 2020 Dec 30;15(12):e0243994. doi: 10.1371/journal.pone.0243994 (PMC7773199; doi:10.1371/journal.pone.0243994)
Supplement: S2 Table — (DOCX) [file pone.0243994.s004.docx]

S2 Table. Balancing test for characteristics before the disaster; results from ordered logistic regression analysis

|  |  | Dependent variable: housing damage^a^ |  |
| --- | --- | --- | --- |
|  | n | Crude OR (95% CI) | P−value |
| Household subjective economic status before the disaster |  |  |  |
| Not stable | 16 | ref | − |
| Fairly stable | 62 | 1.602 (0.500, 5.135) | 0.427 |
| Stable | 76 | 1.353 (0.431, 4.249) | 0.605 |
| Missing | 13 | 1.251 (0.087, 18.023) | 0.869 |
| Mother's education |  |  |  |
| High school or less | 80 | ref | − |
| Some college | 58 | 1.072 (0.562, 2.046) | 0.833 |
| College or more | 16 | 0.394 (0.119, 1.301) | 0.127 |
| Missing | 13 | 0.837 (0.070, 10.055) | 0.888 |
| Father's occupation |  |  |  |
| Non−manual | 30 | ref | − |
| Manual | 86 | 0.899 (0.396, 2.041) | 0.798 |
| Unemployed | 1^b^ | − |  |
| Missing | 46 | 0.66 (0.261, 1.670) | 0.380 |
| Mather's occupation |  |  |  |
| Non−manual | 38 | ref | − |
| Manual | 87 | 0.537 (0.255, 1.131) | 0.102 |
| Unemployed | 23 | 1.168 (0.450, 3.031) | 0.750 |
| Missing | 15 | 1.356 (0.380, 4.841) | 0.639 |
| Exposure to other traumatic experiences before the disaster (ref. no) |  |  |  |
| Involvement in a serious accident | 1^b^ | − |  |
| Witnessed a serious accident | 0 ^b^ | − |  |
| Attacked by a dog or other animals | 0 ^b^ | − |  |
| Had a close friend or family member who had a serious illness | 24 | 0.598 (0.254, 1.410) | 0.240 |
| Death of a close friend or family member | 31 | 1.494 (0.721, 3.094) | 0.280 |
| Visited hospital due to serious disease or injury, or underwent a serious medical procedure, or admitted to hospital | 11 | 0.441 (0.115, 1.688) | 0.232 |
| Separated from a caregiver | 23 | 0.554 (0.217, 1.416) | 0.218 |
| Experienced sexual assault | 0 ^b^ | − |  |
| Experienced other criminal assault | 0 ^b^ | − |  |
| Bullied by peers at preschool or in the neighborhood | 2 ^b^ | 0.869 (0.074, 10.246) |  |
| Experienced violence from a close friend or family member | 4 ^b^ | − |  |
| Witnessed a violent incident involving a close friend or family member | 5 | 0.867 (0.149, 5.033) | 0.874 |
| Had a close friend or family member who attempted suicide | 1 ^b^ | 2.141 (0.121, 37.736) |  |
| Experienced a previous natural disaster Other | 2 ^b^ | 5.343 (0.451, 63.342) |  |
| Other stressful events | 6 | 1.481 (0.308, 7.111) | 0.624 |
| Any of these | 67 | 1.2 (0.650, 2.216) | 0.559 |
| Number of these experiences (continuous) | 0.71^c^ | 0.909 (0.662, 1.248) | 0.554 |

Abbreviations: odds ratio, OR; confidence interval, CI; reference, ref.

a Housing damage was categorized as "no damage," "partly damaged," and "destroyed or flooded."

b OR was not estimated because of the small sample size

c Mean number of trauma experiences before the Great East Japan Earthquake
